# Why Y RNAs? About Versatile RNAs and Their Functions

**DOI:** 10.3390/biom3010143

**Published:** 2013-02-07

**Authors:** Marcel Köhn, Nikolaos Pazaitis, Stefan Hüttelmaier

**Affiliations:** Martin-Luther-University Halle-Wittenberg, Institute of Molecular Medicine, Section Molecular Cell Biology, ZAMED, Heinrich-Damerow-Str.1, D-6120 Halle, Germany; E-Mails: marcel.koehn@medizin.uni-halle.de (M.K.) pazaitis@gmx.de (N.P.)

**Keywords:** Y RNA, Ro60, La, ncRNA

## Abstract

Y RNAs constitute a family of highly conserved small noncoding RNAs (in humans: 83-112 nt; Y1, Y3, Y4 and Y5). They are transcribed from individual genes by RNA-polymerase III and fold into conserved stem-loop-structures. Although discovered 30 years ago, insights into the cellular and physiological role of Y RNAs remains incomplete. In this review, we will discuss knowledge on the structural properties, associated proteins and discuss proposed functions of Y RNAs. We suggest Y RNAs to be an integral part of ribonucleoprotein networks within cells and could therefore have substantial influence on many different cellular processes. Putative functions of Y RNAs include small RNA quality control, DNA replication, regulation of the cellular stress response and proliferation. This suggests Y RNAs as essential regulators of cell fate and indicates future avenues of research, which will provide novel insights into the role of small noncoding RNAs in gene expression.

## 1. Introduction

Over recent years, our view of genomic regulation was severely revised when realizing that we have overseen a yet to be explored plethora of long and short non-coding RNAs (ncRNAs). Various reports revealed that ncRNAs are important regulators of diverse cellular processes. MicroRNAs (miRNAs), for instance, were shown to be important regulators of mRNA-fate in the cytoplasm, where they control translation and turnover of specific target transcripts (reviewed in [1]). On the other hand, long non-coding RNAs can serve as scaffolds for the assembly of subnuclear bodies (e.g., paraspeckles and NEAT1-RNA, [2]). These results reflect just two aspects of the various functions facilitated by ncRNAs, which modulate gene expression at various levels.

Y RNAs were originally identified in the 1980’s by immunopurification with auto-antibodies from patients suffering from systemic lupus erythematosus [3]. The proteins Ro60 (TROVE2, SSA) and La (SSB), which are common auto-antigens in autoimmune diseases (like systemic lupus erythematosus and Sjögren’s syndrome (reviewed in [4]), were identified as the major antigens facilitating the association with these small ncRNAs [5,6,7]. Subsequently, these cytoplasmic ncRNAs were named Y RNAs to distinguish them from nuclear U RNAs, another major class of small ncRNAs in the cell [3,8].

## 2. Structure and Evolution of Y RNAs

**Figure 1:**
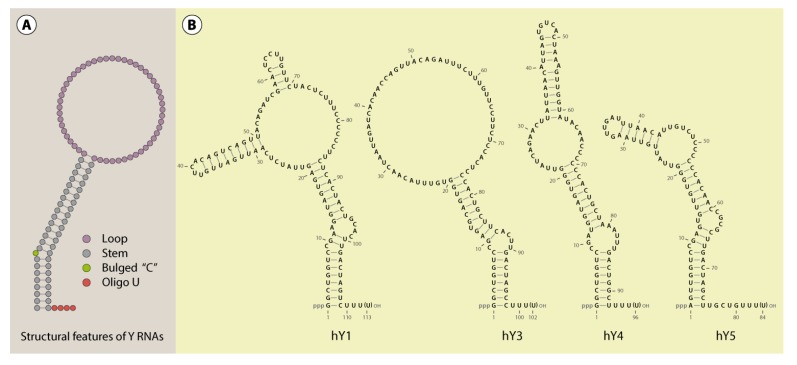
Y RNA structure. A schematic structure of Y RNAs illustrates the important features common to these RNAs (**A**). Furthermore, the secondary structure of the human Y RNAs (Y1, Y3, Y4 and Y5) was visualized with VARNA [17], referring to published structure probing experiments (**B**; [18,19]). According to this data, alternative structures, at least for Y3, are likely [19].

**Figure 2:**
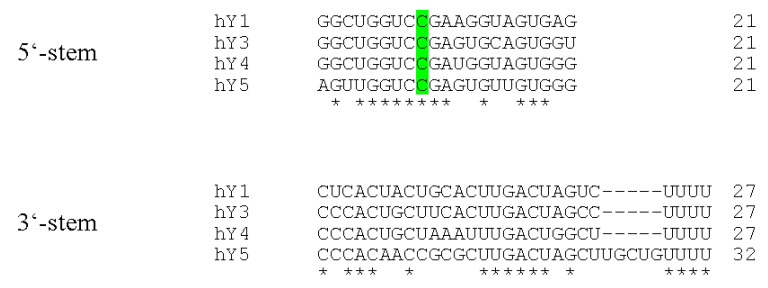
Sequence alignments of human Y RNA stems. The terminal Y RNA stem sequences were aligned using the TCoffee web server [25,26]. Perfectly conserved nucleotides are marked with asterisks. The remarkably conserved cytosine base at position 9 within the 5’-part of the Y RNAs is highlighted in green.

Metazoan Y RNAs are transcribed by RNA polymerase III (POLIII, [9]) from distinct promoters. Transcription is terminated at an OligoT stretch (4–6 nt), resulting in an oligo-uridinylated 3’-end of nascent Y RNA transcripts (Figure 1 and Figure 2). This serves as the primary binding site for the La protein [10,11,12]. It was proposed that the OligoU stretch is removed during nuclear maturation in the nucleus to promote nuclear export of trimmed Y RNAs [13]. The trimming of POLIII-transcripts seems to occur frequently and has been observed for various other RNAs [14]. Consistent with a pivotal role of La in the 3’-end processing of Y RNAs, La-binding was shown to prohibit the transport of Y RNAs to the cytoplasm [15]. However, the molecular basis of Y RNA trimming remains poorly understood and the potentially involved nucleases are still not known. Moreover, Y RNAs were shown to possess triphosphates at their 5’-ends and not to contain large amounts of nucleotide modifications [5].

A common characteristic of all reported Y RNAs is their highly conserved stem-loop structure (Figure 1). The terminal 5’- and 3’-sequences (~20–30 nt) of Y RNAs form a double stranded region - the “stem”. This region is the essential structural determinant of Y RNAs allowing there typical stem-loop fold. For some Y RNAs, these structural properties were validated by enzymatic and chemical cleavage [18,19]. The stems of Y RNAs are usually not perfect double strands. Frequently, the “upper” and “lower” parts are separated by bulged regions. One of these, a highly conserved bulged cytosine (C9 in human Y RNAs [20]), constitutes the primary binding site for the Ro60 protein (TROVE2). Deletion or mutation of this site disrupts Ro60-binding and destabilizes the entire Y RNA fold (Figure 2, [15,20,21]). Ro60-homologs and also Y RNAs have been identified from bacteria to humans, suggesting an ancient origin and co-evolution of the Ro60-Y RNA-complex [21,22,23]. This is supported by analyses confirming the Y RNA stem to be the most conserved part of these ncRNAs, and even the bulged cytosine base in the stem is retained in bacterial Y RNAs (Figure 1 and Figure 2, [23,24]). Accordingly, we propose that organisms, which encode for Ro60 orthologs, presumably also express Y RNAs. Ro60 and its orthologs, as well some Y RNAs, were described or assumed in various bacterial species, lower eukaryotes, like *Chlamydomonas reinhardtii*, nematodes, like *Caenorhabditis elegans*, and vertebrates (reviewed in [21]). Putative Ro60 and Y RNA orthologs had also been suggested in arthropods, such as *Anopheles gambiae* [21,22].

RNA sequencing approaches frequently reported fragments comprising parts of Y RNAs [27,28]. In view of the conserved Y RNA structure resembling that of pre-miRNAs, it accordingly was suggested that Y RNAs could serve as miRNA precursors [28,29]. However, experimental validation of Y RNA encoded regulatory microRNAs is still lacking, and thus, the proposed Y RNA fragments could also result from degradation [30]. In support of this, it was shown that the biogenesis of some Y RNA fragments is independent of DICER1 and AGO2, providing further evidence that the identified fragments are not generated by the classical miRNA pathway [31].

The loops of Y RNAs are heterogeneous in nature and the least conserved of the ncRNAs [24,32]. The primary sequence and length of the loop distinguishes the four Y RNAs (Y1, Y3, Y4 and Y5). The longest loop is observed for Y1 (hY1: 65 nt) and the shortest for Y5 (hY5: 31 nt). The structure of the loops differs significantly among the four Y RNAs and was suggested to be largely flexible in nature [19]. The loops of Y1, Y3 and Y5 are rich in pyrimidines (human RNAs: 69 %, 65 % and 65 %, respectively); only Y1 and Y3 contain large, mostly single stranded stretches of pyrimidines. To date, in *teleostei*, only one Y RNA has been identified, which shows high homology to the human Y1/3-type of Y RNAs. Accordingly, Y3 was suggested to be the most ancestral Y RNA, at least in the vertebrate lineage [24,33]. Furthermore, additional Y RNAs possibly evolved through duplication of this ancestral RNA to fulfill novel functions within cells, which likely involves the loop region [24]. In accordance with this hypothesis, Y RNAs are able to recruit various RNA-binding proteins in a loop-dependent manner (see Table 1).

**Table 1:** Y RNA binding proteins.

Gene Symbol	Alternative Names	Y RNA	Binding Region	Proposed Function	Reference
SSB	La	1,3,4,5	OligoU	nuclear retention, protection of Y RNA 3’ends	[15]
TROVE2	Ro60	1,3,4,5	stem	stabilization,	[15,23,34,35,36,37]
				nuclear export,	
				RNA quality control	
APOBEC3G		1,3,4,5	?	?	[38,39,40]
NCL	nucleolin	1,3	loop	?	[41]
PTBP1	hnRNP I	1,3	loop	?	[42]
HNRNPK		1,3	loop	?	[42]
IGF2BP1	ZBP1, Imp1	(1),3	loop	nuclear Export of Ro60 and Y3	[43,44]
PUF60	RoBP1	(1,3),5	?	?	[45,46]

## 3. Y RNA Localization and Expression

POLIII-transcripts can be transported to the cytoplasm (pre-miRNAs, tRNAs, 5S; [47,48]) or remain in the nucleus after transcription (7SK, U6; [49,50]). The localization of Y1, Y3 and Y4 was described to be mostly cytoplasmic, whereas Y5 seems to be more nuclear [51,52]. As mentioned above, the La protein is thought to interfere with nuclear export of Y RNAs by binding to their 3’-ends [15]. This export block could be released by trimming of Y RNAs, which then lack the OligoU stretch. This would trigger Ro60-dependent nuclear export of these ncRNAs [13,15]. Thus, it is tempting to speculate that Y5 differs in its association with La or nuclear trimming, allowing its nuclear retention. Nascent POLIII transcripts can accumulate in the perinucleolar compartment (PNC, [53,54]). In agreement, at least Y1, Y3 and Y5 were shown to localize to this subnuclear site by fluorescence in *in situ* hybridization (FISH, [55]). In an *in vitro* system, where labeled Y RNAs are incubated with G1 nuclei, Y RNAs were found to associate with euchromatin, and Y5 was recruited to nucleoli [56]. Notably, Y RNAs can be encapsidated into viruses, as shown for Moloney murine leukemia virus (MLV, [13]) and also for human immunodeficiency virus type 1 (HIV-1, [38]). This process is independent of Ro60-binding and seems to be initiated while Y RNAs are still in the nucleus. Whether Y RNAs modulate the lifecycle of these viruses significantly remains unknown. 

The export pathways used by Y RNAs are not known in detail. It was reported that the export of Y RNAs is dependent on the small GTPase Ran, suggesting members of the karyopherin protein family to serve as nuclear export adapters [57]. Although XPO1 and XPOT are presumably not involved, XPO5 seems to be important to direct cytoplasmic translocation of Y RNAs [15,57]. This protein usually exports minihelix containing dsRNAs, which includes VA1, some tRNAs and pre-miRNAs [58,59]. The Y RNA stem is reminiscent of a minihelix, and consistently, XPO5 was shown to associate in a complex with Y1 and RanGTP [58]. This was also supported by the crystal structure of XPO5, indicating the Y RNA stem acts as a substrate for this karyopherin [47]. Notably, there is no evidence for a re-import of Y RNAs into the nucleus. This is supported by the complete nuclear export of radiolabeled Y RNAs after injection into *Xenopus* oocytes [15,57]. Notably, the subcellular localization of Y RNAs was reported to be cell cycle-dependent and respond to cellular stress signals, like UV-irradiation [23,56,60]. Accordingly, cells accumulate both Ro60 and Y RNAs in the nucleus after UV irradiation or oxidative stress [35,44,60]. This could result from the stress-induced collapse of the Ran gradient and concomitant impairment of nuclear export [61], but may furthermore imply stress-dependent functions of the nuclear Ro60-Y RNA-complex under these conditions.

**Figure 3:**
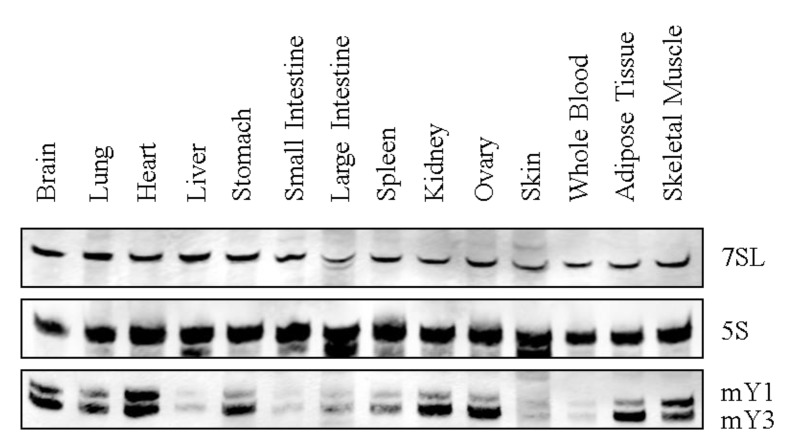
Y RNA expression in mouse tissues. A representative Northern blot for murine Y RNA tissue expression is shown. 7SL and 5S rRNA served as a loading control. Note that in mice, just Y1 and Y3 are expressed.

Y RNA expression has been reported in various species, including primary tissue and tumor-derived cell lines [62,63]. However, comprehensive analyses of tissue-specific Y RNA expression profiles are still lacking. Therefore, we analyzed the expression of murine Y RNAs in several adult tissues by Northern blotting (Figure 3). These studies revealed basal expression of murine Y RNAs (mY1 and mY3) in all analyzed tissues. Y RNA abundance varied significantly, with high levels observed in the brain, lung, heart, stomach, kidney, ovary, adipose tissue and skeletal muscle, in contrast to lower mY RNA abundance in the liver, gut, spleen, skin and blood. Intriguingly, the observed mY RNA expression pattern correlated to the expression signature of Ro60 [64]. This supports the view that the Y RNA-Ro60 complex co-evolved and that the protein is essential for Y RNA stabilization [34,36]. In humans, Y RNAs were shown to be significantly upregulated in a variety of tumors, for instance, bladder and kidney carcinomas. Moreover, Y RNAs promote cell proliferation, which is supported by reduced cell cycle progression upon siRNA-directed Y1 and Y3 depletion [63,65]. Little is known about the developmental expression of Y RNAs, which was analyzed exclusively in *Xenopus laevis* and *Danio rerio*, where Y RNA levels increased after the midblastula transition (MBT, [66]). Notably, the inhibition of Y RNAs by antisense morpholinos led to lethal developmental defects after MBT before gastrulation. 

## 4. Is the Functional Role of Y RNA Determined by Associated Proteins?

### 4.1. Y RNP Core Proteins

All metazoan Y RNAs associate with Ro60 and La, which presumably form the core of nuclear or cytoplasmic Y RNPs (RNP: Ribonucleoprotein). As mentioned above, La binds the OligoU-stretch at the 3’-end of nascent Y RNAs [12]. On the contrary, Ro60 binds to the Y RNA stem [7]. Although La was found at the transcription site of Y RNAs, it remains to be addressed whether the protein has any influence on the transcription process itself [67]. Most likely, the protein protects nascent Y RNA transcripts from being degraded by 3’-exonucleases in the nucleus. Ro60, on the other hand, stabilizes the Y RNA structure and prevents Y RNA degradation [34,36]. In agreement, Y RNA levels are severely reduced in Ro60^−/^^−^ cells [35]. However, the stabilizing function of Ro60 is presumed to mainly affect the cytoplasmic pool of Y RNAs, as shown for mouse fibroblasts [13]. Hence, it remains elusive whether other factors facilitate a Ro60-like role in modulating Y RNA fate in the nucleus [13,68]. Notably, the stabilization of Y RNAs by Ro60 is conserved in the bacterium *Deinococcus radiodurans*, where they act as essential facilitators of UV-resistance [23]. Additionally, the bacterial Rsr (Ro sixty related) was shown to assist in 23S rRNA maturation, and Y RNA were reported to sequester Rsr to inhibit this activity [37]. The crystal structure of Ro60-Y RNA complex revealed that Y RNAs associate with the outer surface of the HEAT-repeat-ring of Ro60, which comprises a highly conserved histidine residue (H187 in human and mouse) that is essential for direct contact formation with Y RNAs [69]. This association surface partially overlaps with the binding site for misfolded ncRNAs (like 5S), implying that Y RNAs modulate the proposed role of Ro60 in the quality surveillance of ncRNAs [60,69,70,71]. 

All four human Y RNAs associate with the antiviral cytidine deaminase APOBEC3G, which accordingly was observed in Ro60- and La-RNPs [38,39,40]. Mutational inactivation of the zinc-binding domain of APOBEC3G (W127A) strongly reduced its interaction with Y1 [38]. Hence, although the function of APOBEC3G-Y RNA complexes remains unknown, one could speculate that the protein facilitates C-to-U-editing to modulate Y RNA-functions and/or suppress retro-transposition of these ncRNAs [39]. Notably, retro-pseudogenes derived from Y RNAs have been described, but it is unclear if they can be re-expressed or have any other functional relevance [72]. The existence of another putative Y RNA core protein, which binds the upper Y RNA stem, is still controversial. Based on sequence requirements, this protein could be involved in nuclear export of Y RNAs and the proposed involvement of Y RNAs in DNA-replication [57,73]. *In vitro*, all four human Y RNAs were reported to associate with proteins of the pre-replication, as well as the origin recognition complex (e.g., ORC2 and CDT1, [56,66]). However, the functional composition of these Y RNPs remains to be deciphered, since the Y RNP core proteins, Ro60 and La, as well as nucleolin association, are not a prerequisite for Y RNAs to function in DNA replication [74]. 

### 4.2. Accessory Y RNA Binding Proteins

**Figure 4:**
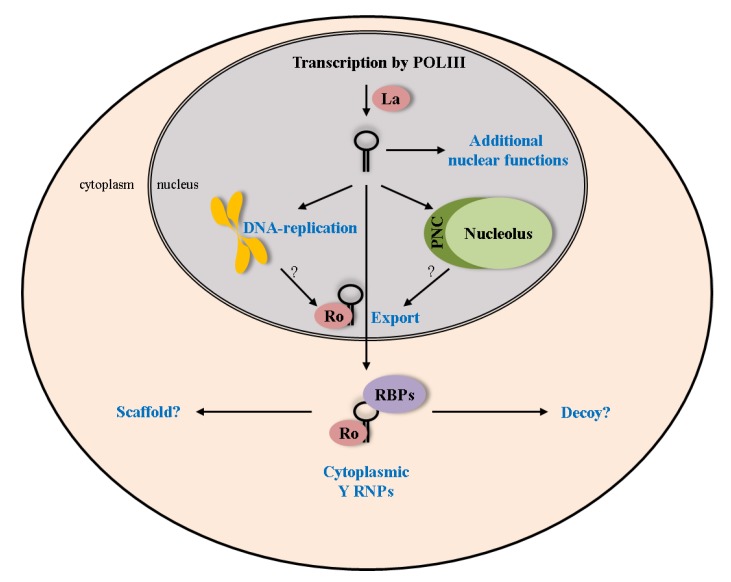
Lifecycle of Y RNAs. The proposed cellular functions of Y RNAs rely mostly on the association with their core proteins Ro60 and La. These interactions influence various parts of the Y RNA lifecycle (e.g., nuclear export together with Ro60). Additional functions and interactions with RNA binding proteins (RBPs) have to be assumed in the nucleus, as well as the cytoplasm.

Apart from proteins associating at the Y RNA stem, additional proteins have been shown to bind Y RNAs. Most of these associate with distinct Y RNA loops and, thus, may impose Y RNA specific functions or play a role in directing the potentially distinct lifecycle of each Y RNA (for a summary, see Table 1 and Figure 4). Only the Y1- and Y3-RNAs contain pyrimidine-rich tracts within their loops. Accordingly, these Y RNAs associate with OligoU/C-binding proteins, including hnRNP I (PTBP1), hnRNP K (HNRNPK) and Nucleolin (NCL) [41,42]. Additionally, RoBP1 (PUF60) was shown to bind Y5 and also associates with Y1 and Y3, *in vivo* and *in vitro* [45,46]). The purification procedure used for PUF60-Y5-complexes led to the identification of RPL5 as a putative binding partner of Y5. In support of this, Y5 was shown to associate with 5S-rRNA-variants, suggesting a role of this Y RNA in the biogenesis of ribosomal RNAs [46], as demonstrated for Ro60 and La [75,76]. The identification of Ro60 in IGF2BP1 containing RNPs suggested an association of this protein with Y RNAs [77]. Recently, we and the Wolin lab could confirm this by demonstrating that mouse IGF2BP1, like its chicken ortholog ZBP1 (Zipcode binding protein), directly associates with Y3 and, to a lesser extent, with Y1 [43,44]. The depletion of IGF2BP1 resulted in the nuclear accumulation of Ro60 and Y3, suggesting an involvement of the protein in nuclear export of this Ro60-Y RNA complex [44].

Despite the various proteins described to associate with Y RNAs, the composition and properties of cellular Y RNPs remains largely elusive. Gel filtration studies indicate that Y RNPs range in size from 150–550 kDa. This suggests that one Y RNA can associate with more than one protein simultaneously. *In vitro*, we and others observed ternary complexes comprising La, Y3 and ZBP1 [43,78]. These findings suggest that Y RNPs contain at least one Y RNA, one core protein (e.g., Ro60) and one or two directly loop-associated accessory proteins (e.g., ZBP1), which could serve as binding scaffolds for additional proteins or promote oligomerization of Y RNPs (Figure 4).

## 5. Future Perspectives and Conclusions

Although various Y RNA-associated proteins have been reported, the role of this highly conserved family of small ncRNAs and, in particular, the reason for their diversification remains sparse. Only two Y RNA functions have been proposed so far. During the cell cycle, they were suggested to stimulate DNA replication (Figure 4; [65,73,79]). This assumption is mainly based on *in vitro* evidence, using isolated G1-phase nuclei incubated with cellular extracts. These studies also indicated that Y RNAs are required for the establishment of new replication forks, but not for DNA elongation [79]. Although the model of Y RNAs being involved in DNA replication is intriguing, more information regarding the molecular mechanisms and *in vivo* regulation of these processes are required. However, a role of Y RNAs in DNA replication could be supported by their high conservation, as well as the devastating developmental defects and the proliferation decrease upon Y RNA inhibition [63,66]. On the other hand, Y RNAs were reported to inhibit the function of Ro60 in RNA quality control [37]. Accordingly, it was also shown that the binding site for Y RNAs in Ro60 partially overlaps with the one for misfolded RNAs, suggesting a mutually exclusive Ro60-RNP [69]. Therefore, we favor a model that Y RNAs and bound Ro60 act as cellular stress sensors. Accordingly, Ro60 can dissociate from Y RNAs under conditions, like UV-irradiation, to assist in cellular recovery by salvaging misfolded RNAs (reviewed in [21]). We propose that, in addition to Ro60, other RBPs could be sequestered by Y RNAs in a similar fashion. This is in accordance with the proposed functions of long ncRNAs, which can act as decoys and/or scaffolds to regulate gene expression [80]. A putative scaffolding role could provide both a sequestering of these regulators acting like a “molecular sink” or a chaperoning function to expedite function of the associated proteins [80]. We thus expect that Y RNAs modulate additional regulatory processes controlling the fate of mRNAs, which could include RNA processing, as well as cytoplasmic regulation of gene expression. The latter assumption is supported by the observation that Y RNAs are predominantly cytoplasmic at steady state. Surprisingly, most of the proposed roles of Y RNAs involve nuclear functions like DNA-replication or small RNA quality control. Future work will thus have to address the compartment-specific function of Y RNPs, which we expect to reveal that these small ncRNAs serve functions in modulating cytoplasmic mRNA fate. This role could essentially rely on the capability of Y RNAs to associate with various RBPs observed in mRNPs, for instance, IGF2BP1 (reviewed in [81]). By controlling the accessibility of such factors, Y RNAs could modulate the function of these regulatory RBPs in mRNA turnover, translation and, potentially, mRNA localization at regular or stress conditions. In addition to revealing the cellular role of Y RNAs, future studies also have to address the physiological significance of these small ncRNAs by the use of genetic models to address their function in development and diseases. 

## 6. Materials and Methods: Isolation of Total RNA and Northern Blot

Tissues were isolated from athymic Nude-Foxn1^nu^-mice (Harlan) and rapidly mixed with TRIZOL-reagent (Life Technologies). RNA-extraction was then performed using chloroform and precipitation with isopropanol. For Northern Blotting, 2.5 µg of total RNA was resolved on a 15% denaturing TBE-Urea-gel and subsequently blotted onto nylon membranes (Roche). The membranes were then UV-crosslinked (Stratalinker 2400) and pre-hybridized with PerfectHyb Plus (Sigma-Aldrich). Northern probes (Atto680 or DY-782 label) were diluted to 100 ng/µL in PerfectHyb Plus and hybridized at 30 °C for 2 hours. Detection was conducted using the Odyssey Scanner (LI-COR). Northern Probes: 7SL: GGCATAGCGCACTACAGCCCAGAACTCCTG; Y1: ATAACTCACTACCTTCGGACCAGCC; Y3: CTGTAACTGGTTGTGATCAATTAGT; and 5S: AAGTACTAACCAGGCCCGAC.
